# Receptor for Advanced Glycation End Products (RAGE) and Its Ligands: Focus on Spinal Cord Injury

**DOI:** 10.3390/ijms150813172

**Published:** 2014-07-25

**Authors:** Juhyun Song, Won Taek Lee, Kyung Ah Park, Jong Eun Lee

**Affiliations:** 1 Department of Anatomy, Yonsei University College of Medicine, Seoul 120-752, Korea; E-Mails: sjh1008@yuhs.ac (J.S.); inskull@yuhs.ac (W.T.L.); kapark@yuhs.ac (K.A.P.); 2 BK21 Plus Project for Medical Sciences, and Brain Research Institute, Yonsei University College of Medicine, Seoul 120-752, Korea

**Keywords:** receptor for advanced glycation end products (RAGE), spinal cord injury (SCI), inflammation, neurite outgrowth, Schwann cell

## Abstract

Spinal cord injury (SCI) results in neuronal and glial death and the loss of axons at the injury site. Inflammation after SCI leads to the inhibition of tissue regeneration and reduced neuronal survival. In addition, the loss of axons after SCI results in functional loss below the site of injury accompanied by neuronal cell body’s damage. Consequently, reducing inflammation and promoting axonal regeneration after SCI is a worthy therapeutic goal. The receptor for advanced glycation end products (RAGE) is a transmembrane protein and receptor of the immunoglobulin superfamily. RAGE is implicated in inflammation and neurodegeneration. Several recent studies demonstrated an association between RAGE and central nervous system disorders through various mechanisms. However, the relationship between RAGE and SCI has not been shown. It is imperative to elucidate the association between RAGE and SCI, considering that RAGE relates to inflammation and axonal degeneration following SCI. Hence, the present review highlights recent research regarding RAGE as a compelling target for the treatment of SCI.

## 1. Introduction

Spinal cord injury (SCI) is considered to be primarily associated with loss of motor function [1] and leads to activate diverse cellular mechanisms in the central nervous system (CNS) to attempt to repair the damaged spinal cord tissue [2]. Potential treatments for SCI, including stem cell therapy [3,4], transplantation of Schwann cells [5,6], neurotrophin and growth factor delivery [7,8], and regulation of inflammatory responses in injured spinal cord [9,10] have recently been investigated. SCI provokes an inflammatory response that causes further tissue damage and neurodegeneration [11]. The inflammation following SCI is considered an important process that promotes secondary damage to neuronal tissue in the spinal cord after traumatic injury and regulates the pathological progress during SCI [12,13,14,15]. After SCI, apoptotic cell death is observed in neurons and oligodendrocytes. In addition, Wallerian degeneration of white matter is simultaneously observed [16,17,18]. The receptor for advanced glycation end products (RAGE), a transmembrane protein and member of the immunoglobulin superfamily, is expressed in endothelial cells, neurons, macrophages, and monocytes [19]. RAGE binds diverse ligands, such as high mobility group box-1 (HMGB1) [20] and S100β [21], and is implicated in various diseases [22,23,24,25,26,27,28,29,30]. In this review, we will focus on how RAGE and its ligands are involved in various mechanisms that are activated following SCI. Specifically, we focus on three points: (1) the association between RAGE and inflammation following SCI; (2) the association between RAGE and neurite outgrowth after SCI; and (3) the association between RAGE and Schwann cell growth after SCI. Here, we highlight recent research regarding the rationale behind choosing RAGE and its ligands as potential targets for treating SCI. 

## 2. Spinal Cord Injury

**Figure 1:**
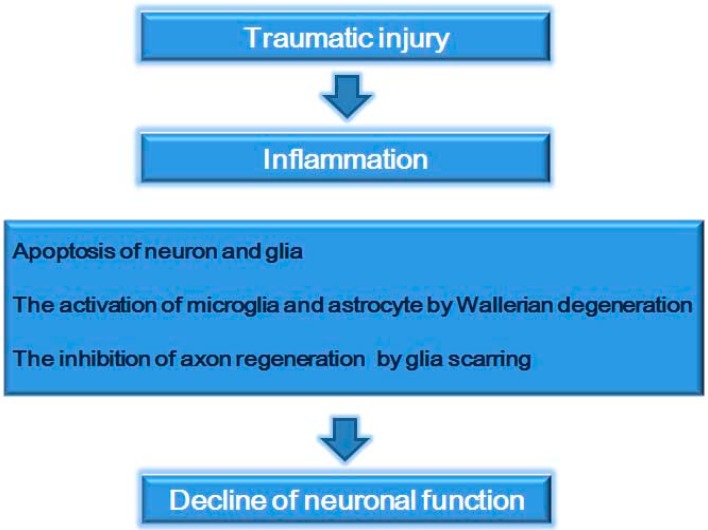
Schematic representation regarding various responses following spinal cord injury. Spinal cord injury caused by traumatic mechanical injury leads to the secondary damages including inflammatory response, Wallerian degeneration, the activation of glia cells, glia scar formation. Finally, these secondary damages cause decline of neuronal function following spinal cord injury.

SCI resulting from mechanical trauma provokes secondary injuries, including severe tissue damage, inflammation, Wallerian degeneration, disruption of the blood-spinal cord barrier, myelin degradation, and glial scarring [2,31,32,33] (Figure 1). SCI has been reported to have an increased annual incidence [34]. Functional recovery due to the repair of the injured spinal cord is a major challenge in the neuroscience research field [35]. After SCI, inflammation is a major response and source of secondary injury, considering that it modulates the pathogenesis of acute and chronic SCI [36]. Inflammatory responses cause apoptosis of neurons and glia, as well as glial scar formation and the decline of neuronal function [37]. Pro-inflammatory cytokines, such as tumor necrosis factor-alpha (TNF-α) and interleukin-1 beta (IL-1β), are expressed by CNS cells, including microglia, astrocytes, neurons, and oligodendrocytes at early time points (one to three hours) after SCI [38,39,40]. In the injured spinal cord, increased TNF-α and IL-1β promote vascular permeability, inflammation, release of pro-inflammatory cytokines, and apoptosis of oligodendrocytes and neurons [41,42,43,44,45,46]. In addition, resident microglia are activated near the injury site with subsequent recruitment of neutrophils, macrophages, and lymphocytes after SCI [47]. Subsequently, Wallerian degeneration triggers the activation of microglia and astrocytes in injured spinal cord tissues [48,49]. Glial scarring from SCI is considered an obstacle for axon regeneration [50,51] and is an inducer of inflammatory cascades [52]. At the site of injury and inflammation, axons are engulfed by macrophages [53]. Subsequently, these direct macrophage–axon interactions can promote axonal degeneration [53]. Several studies have reported that inflammation caused by SCI contributes to neuronal degeneration and loss of motor function below the injury site [54,55,56]. Consequently, inflammation should be modulated to reduce secondary injuries and functional decline following SCI. Hence, the study on the mechanisms of inflammation following SCI is important to find the therapeutic solution in SCI. 

## 3. RAGE

RAGE is a 35-kDa membrane-bound protein receptor and a member of a superfamily of immunoglobulins [57] and shares homology with neuronal cell adhesion molecules such as NCAM or axonin [58,59]. RAGE consists of an extracellular moiety which is required for signal transduction and includes one *N*-terminal V-type and two C-type Ig domains [60]. RAGE was originally recognized as a receptor of advanced glycation end products (AGEs), which accumulate in diabetes and during aging [61]. Numerous studies demonstrate that AGEs form during metabolic processes involving proteins or peptides and sugars. AGEs are major sources of diabetic complications, such as atherosclerosis [25], nephropathy [27,62,63], and inflammation [26,64,65] due to their interactions with RAGE. RAGE binds to AGEs [66,67], but can also bind to other ligands, including a HMGB1 (amphoterin) [66,67,68,69,70], β-amyloid fibrils [28,71,72,73,74], and S100 proteins [24,75,76,77]. The RAGE VC1 domain has a net positive charge, but the C2 domain has a net negative charge [78,79]. The negatively charged HMGB1 *C*-domain [80,81], AGEs, and S100 proteins are attracted to the positive charge of the VC1 domain [78,79]. Recent research demonstrates that RAGE is involved in diseases, including CNS diseases, due to interactions with these various ligands [22,23,24,25,26,27]. RAGE has been reported to induce transduction pathways involving Ras (related with apoptosis through interaction with AGE in response to oxidative stress) [82], Rac/Cdc42 (related with neurite outgrowth through binding with amphoterin) [66], Jak/signal transducer and activator of transcription (Jak/STAT) (related with alteration of gene expression/cytokine production through interation with HMGB1) [22,23,24,25,26,64,83,84], extracellular signal-regulated kinase (ERK) (related with cell survival/cell proliferation with activation of Rac-1 and Cdc42) [85,86,87], nuclear factor κ-light-chain-enhancer of activated B cells (NF-κB) (related with cell apoptosis/cytokine production) [69,88,89,90]. RAGE is involved in various inflammatory mechanisms and participates in numerous diseases including CNS disorders by binding diverse ligands. In particular, several studies reported that RAGE was upregulated after SCI in rats and mice [91,92]. Also, by confirming using RAGE deficiency animals, RAGE was demonstrated that it plays a cardinal role in the various pathophysiological process of SCI [93]. Hence, RAGE should be examined to understand the mechanisms leading to secondary damage after SCI, particularly because RAGE is associated with inflammatory responses following SCI.

## 4. RAGE and Its Ligands (HMGB1 and S100β): Focus on Inflammation Following SCI

**Figure 2:**
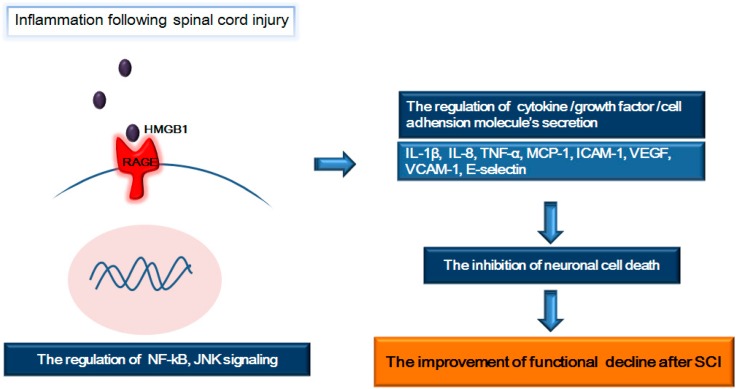
Schematic representation regarding the role of receptor for advanced glycation end products (RAGE) in inflammation following spinal cord injury. After spinal cord injury, RAGE binds to high mobility group box-1 (HMGB1) and subsequently follows in the nuclear factor kappa-light-chain-enhancer of activated B cells (NF-κB) or JNK pathway. The interaction RAGE and HMGB1 regulates the production of a variety of cytokines, adhesion molecules and growth factors and finally inhibits neuronal cell death. Thus, the interaction RAGE and HMGB1 may improve the functional decline after spinal cord injury.

Among RAGE’s ligands, HMGB1 and S100β protein should be focused on the SCI’s research because these ligands participate in secondary mechanisms after SCI along with RAGE. RAGE is a major cellular binding site for HMGB1 (amphoterin) [57,94,95]. It acts as a pattern recognition receptor and participates in the innate immune response [61,96]. HMGB1 is generally expressed in cellular nuclei and cytoplasm in brain regions, including the hippocampal dentate gyrus, olfactory bulbs, cell lining of the telencephalic ventricles [97], the nuclei of adult neurons and astrocytes [98], spinal cord oligodendrocytes [99], choroid plexus endothelial cells [100], microglia [101], and Schwann cells [102]. S100β is abundantly expressed in astrocytes and binds to RAGE in the CNS [21]. HMGB1 and S100β protein has been reported to modulate both beneficial and harmful effects in *in vitro* study [103,104]. The interactions of RAGE with these ligands activate diverse mechanisms, including inflammation, oxidative stress, neurodegeneration, promotion of neurite outgrowth, cell survival, and neuronal differentiation [20,60,67,68,105,106]. Several studies demonstrate that mechanical injury following SCI results in secondary injuries, such as inflammatory responses [12,107], hemorrhage, ischemia, excessive free radical generation, vascular dysregulation, and immune cell infiltration [108,109,110]. Specifically, inflammation following SCI has been known to play an important role in the regulation of remyelination and neuronal and glial cell death [12,15,107,111,112,113,114,115]. Inflammation has been recognized as an important process that affects the progression of neuronal tissue damage following SCI [15,116,117]. Cell death caused by inflammation is affected by injury-promoting factors, such as pro-inflammatory cytokines [15,92]. Within one hour post-SCI, TNF-α and interleukin-6 (IL-6) are strongly upregulated around the contused site [38,118]. Inhibition of cell death is important for improving neurologic dysfunction following SCI [15,92]. Inflammation during SCI has been regarded as be important the regulation through NF-κB [119]. A transgenic mouse model of SCI, in which NF-κB was selectively suppressed in astrocytes, showed reduced inflammation and increased axonal sprouting [120,121]. RAGE activation perpetuates NF-κB p65 activation by de novo synthesis of NF-κB p65 which is directly associated with cell proliferation and that the interaction of RAGE and HMGB1 increases the expression of NF-κB p65 [122]. Among the ligands of RAGE, HMGB1 is specifically associated with neuronal cell death following SCI [15,91]. HMGB1 has been found to be elevated in injured spinal cord tissues of rodents [92,123,124,125]. HMGB1 binds to RAGE on neurons, glia, and endothelial cells in the CNS [126,127,128]. HMGB1 in living cells resides [129] mostly in the nucleus whereas necrotic cells release HMGB1 immediately [130,131]. HMGB1 accelerates inflammatory responses through the binding with RAGE [20,132,133,134]. RAGE contributes to inflammatory responses [135] by regulating the production of cytokines, such as interferon-gamma (IFN-γ) [136], interleukin-6 (IL-6), and TNF-α [137,138], IL-1β [139] in monocytes and macrophages [140,141,142] after binding with HMGB1. In addition, interaction of RAGE and HMGB1 regulates the production of chemokines [143,144] to activate the immune cells such as dendritic cells [145,146] and monocytes [147]. Moreover, the binding of HMGB1 to RAGE regulates the migration of immune cells and the upregulation of interleukin-8 (IL-8), monocyte chemotactic protein-1 (MCP1), vascular endothelial growth factor (VEGF), intercellular adhesion molecule-1 (ICAM-1), vascular cell adhesion molecule-1 (VCAM-1), and E-selectin [133,148,149,150]. Subsequently, the binding of HMGB1 to RAGE participates in neovascularization after injury [133,148,149,150]. In addition, HMGB1-induced signaling through RAGE activates diverse signaling pathways, such as the JNK and NF-κB pathways [66,149], in inflammatory environments. The binding of HMGB1 to RAGE seems to contribute to the inflammatory response generating the secondary damage of SCI by controlling the secretion of cytokine and chemokine and by mediating apoptosis signaling (Figure 2).

## 5. RAGE and Its Ligands: Focus on Neurite Outgrowth Following SCI

**Figure 3:**
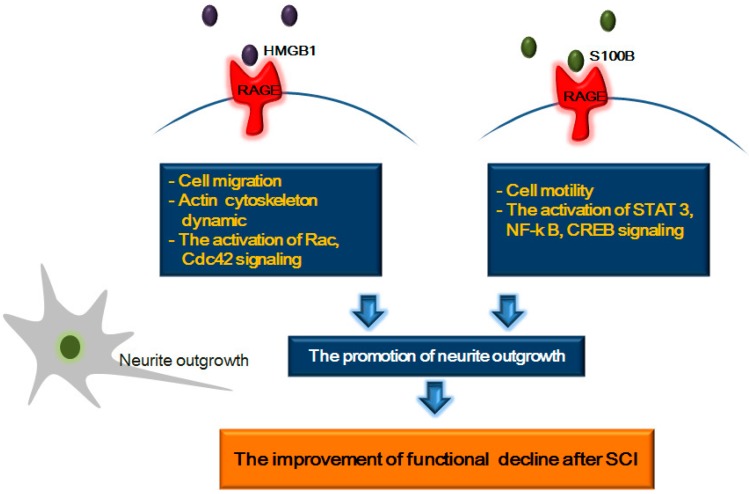
Schematic representation of the relationship between RAGE and neurite outgrowth after spinal cord injury. The binding RAGE to HMGB1 promotes Rac, Cdc42 pathway and is involved in the dynamics of actin cytoskeleton. Also, the binding RAGE to S100B increases cell motility and is related to CREB, NF-κB, STAT3 signaling. These mechanisms result in the promotion of neurite outgrowth and finally may improve the functional decline after spinal cord injury.

SCI results in the failure of axonal regeneration, leading to functional decline [151]. To improve function after SCI, various solutions, such as neural precursor cell transplantation to increase remyelination, have been proposed [152,153,154,155,156,157]. Axon regeneration and neurite outgrowth are key to treating functional decline following SCI. Several studies demonstrate that HMGB1 promotes neurite outgrowth, cell migration after injury [68,92,101,158]. Binding of amphoterin to RAGE has been known to promote neurite outgrowth [20,66,68,104,159,160]. HMGB1 binds to RAGE-associated *N*-glycans and subsequently promotes neurite outgrowth [161,162]. Hori *et al.* demonstrated that anti-RAGE IgG/(Fab')2 inhibited HMGB1-RAGE activation and blocked HMGB1-induced neurite outgrowth [20]. Several studies demonstrated that RAGE-amphoterin interaction involves Rac and Cdc42, and promotes the neurite outgrowth [66,163]. In addition, several studies show the role of RAGE in cell migration [143,144,164,165,166,167] and in the dynamics of the actin cytoskeleton [143]. RAGE signaling mediates neurotrophin-dependent neurite outgrowth [168]. Among the RAGE ligands, S100β also has known to promote neurite outgrowth and induce translocation of transcription factors, such as NF-κB and CREB, following interaction with RAGE [21,104,169,170,171]. In detail, S100β-RAGE activation regulates neurite outgrowth through STAT3 and p44/p42 MAP kinases via RAGE [172]. S100β-mediated RAGE activation also participates in cell motility [164]. In a study on the function of RAGE in cell motility, transfectants exposed to amphoterin induced neuritis [66]. Collectively, the interaction between RAGE and HMGB1/S100β may activate cell motility and neurite outgrowth after SCI (Figure 3). Hence, RAGE and its ligands may improve functional decline after SCI by promoting neurite outgrowth, considering that regulation of neurite outgrowth is important for neurite regeneration after nerve injury [173].

## 6. RAGE and Its Ligands: Focus on Schwann Cell Growth and Migration after SCI

**Figure 4:**
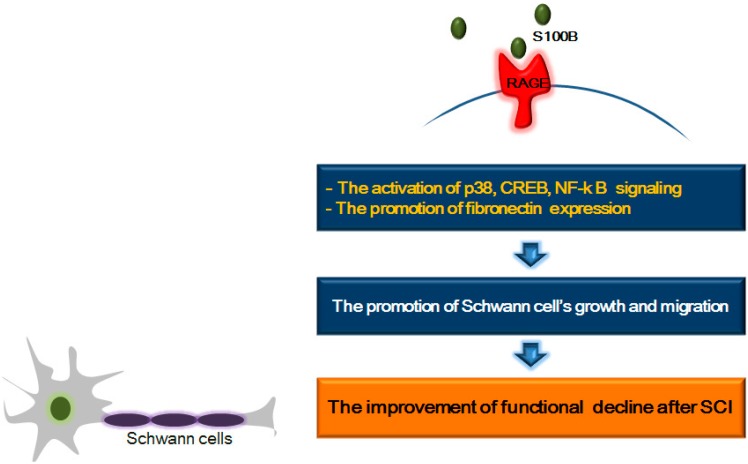
Schematic representation of the association between RAGE and Schwann cell growth and proliferation after spinal cord injury. After spinal cord injury, the interaction RAGE and S100β promotes the activation of NF-κB, CREB, p38 signaling and the expression of fibronectin. These mechanisms may promote the growth and proliferation of Schwann cells and finally improve the functional decline after spinal cord injury.

Schwann cells have important roles in tissue repair after CNS injury because Schwann cells are involved in various mechanisms, including differentiation, migration, proliferation, and myelination of axons [174,175]. In addition, Schwann cells promote the secretion of a variety of neurotrophic factors, subsequently inducing axonal regeneration after CNS injury [174]. After SCI, Schwann cells are sometimes observed in remyelinating axons in the spinal cord [110,176,177,178,179]. The remyelination by Schwann cells in the spinal cord is regarded as the result of migration from the periphery [176]. Wiliams *et al.* reported that the implantation of Schwann cells in injured spinal cords supports regeneration of axons, reduces cyst formation, and improves functional decline [180]. Schwann cell myelination occurs through increased fibronectin expression [181]. S100β-mediated RAGE activation promotes mRNA expression of fibronectin [182,183]. Several studies demonstrated that Schwann cells, RAGE, and S100β are necessary for peripheral nerve regeneration [182,183,184]. RAGE plays a key role in Schwann cell’s function during regeneration of injured nerves [185]. Moreover, S100β-activated RAGE has been reported the association with Schwann cell migration during the repair procedure of injured peripheral nerves through activation of p38 MAPK, CREB, and NF-κB [186]. These researches indicate that the interaction between RAGE and its ligands may promote the Schwann cell’s beneficial function including secretion of neurotrophic factors, myelination of axons and axonal regeneration for treating after spinal cord injury (Figure 4). 

## 7. Conclusions

In the present review, we discuss the association of RAGE and its ligands with SCI from various perspectives. We addressed three points: (1) Binding of RAGE and its ligands appears to contribute to the inflammatory response caused by SCI by regulating the secretion of cytokines and chemokines and modulating apoptosis signaling; (2) RAGE and its ligands may improve functional decline after SCI by promoting neurite outgrowth, which is crucial for neurite regeneration after CNS injury; (3) RAGE and its ligands may promote the myelination of axons and axonal regeneration as treatments for injured spinal cords by activating Schwann cells. This review indicates that further studies of RAGE and its ligands in SCI are necessary for a good understanding of the various mechanisms leading to functional decline after SCI. 

## References

[1] Scott J.M., Warburton D.E., Williams D., Whelan S., Krassioukov A. (2011). Challenges, concerns and common problems: Physiological consequences of spinal cord injury and microgravity. Spinal Cord.

[2] Thuret S., Moon L.D., Gage F.H. (2006). Therapeutic interventions after spinal cord injury. Nat. Rev. Neurosci..

[3] Teng Y.D., Lavik E.B., Qu X., Park K.I., Ourednik J., Zurakowski D., Langer R., Snyder E.Y. (2002). Functional recovery following traumatic spinal cord injury mediated by a unique polymer scaffold seeded with neural stem cells. Proc. Natl. Acad. Sci. USA.

[4] Lopez-Gonzalez R., Velasco I. (2012). Therapeutic potential of motor neurons differentiated from embryonic stem cells and induced pluripotent stem cells. Arch. Med. Res..

[5] Pearse D.D., Marcillo A.E., Oudega M., Lynch M.P., Wood P.M., Bunge M.B. (2004). Transplantation of Schwann cells and olfactory ensheathing glia after spinal cord injury: Does pretreatment with methylprednisolone and interleukin-10 enhance recovery?. J. Neurotrauma.

[6] Oudega M., Xu X.M. (2006). Schwann cell transplantation for repair of the adult spinal cord. J. Neurotrauma.

[7] Hendriks W.T., Ruitenberg M.J., Blits B., Boer G.J., Verhaagen J. (2004). Viral vector-mediated gene transfer of neurotrophins to promote regeneration of the injured spinal cord. Prog. Brain Res..

[8] Taylor S.J., McDonald J.W., Sakiyama-Elbert S.E. (2004). Controlled release of neurotrophin-3 from fibrin gels for spinal cord injury. J. Control. Release.

[9] Bethea J.R., Nagashima H., Acosta M.C., Briceno C., Gomez F., Marcillo A.E., Loor K., Green J., Dietrich W.D. (1999). Systemically administered interleukin-10 reduces tumor necrosis factor-alpha production and significantly improves functional recovery following traumatic spinal cord injury in rats. J. Neurotrauma.

[10] Gok B., Sciubba D.M., Okutan O., Beskonakli E., Palaoglu S., Erdamar H., Sargon M.F. (2009). Immunomodulation of acute experimental spinal cord injury with human immunoglobulin G. J. Clin. Neurosci..

[11] Fleming J.C., Norenberg M.D., Ramsay D.A., Dekaban G.A., Marcillo A.E., Saenz A.D., Pasquale-Styles M., Dietrich W.D., Weaver L.C. (2006). The cellular inflammatory response in human spinal cords after injury. Brain.

[12] Nakahara S., Yone K., Sakou T., Wada S., Nagamine T., Niiyama T., Ichijo H. (1999). Induction of apoptosis signal regulating kinase 1 (ASK1) after spinal cord injury in rats: Possible involvement of ASK1-JNK and -p38 pathways in neuronal apoptosis. J. Neuropathol. Exp. Neurol..

[13] Liu S., Ruenes G.L., Yezierski R.P. (1997). NMDA and non-NMDA receptor antagonists protect against excitotoxic injury in the rat spinal cord. Brain Res..

[14] Kato H., Kanellopoulos G.K., Matsuo S., Wu Y.J., Jacquin M.F., Hsu C.Y., Kouchoukos N.T., Choi D.W. (1997). Neuronal apoptosis and necrosis following spinal cord ischemia in the rat. Exp. Neurol..

[15] Crowe M.J., Bresnahan J.C., Shuman S.L., Masters J.N., Beattie M.S. (1997). Apoptosis and delayed degeneration after spinal cord injury in rats and monkeys. Nat. Med..

[16] Mattson M.P. (2000). Apoptosis in neurodegenerative disorders. Nat. Rev. Mol. Cell Biol..

[17] Mizuno Y., Mochizuki H., Sugita Y., Goto K. (1998). Apoptosis in neurodegenerative disorders. Intern. Med..

[18] Byrnes K.R., Stoica B.A., Fricke S., di Giovanni S., Faden A.I. (2007). Cell cycle activation contributes to post-mitotic cell death and secondary damage after spinal cord injury. Brain.

[19] Stern D., Yan S.D., Yan S.F., Schmidt A.M. (2002). Receptor for advanced glycation endproducts: A multiligand receptor magnifying cell stress in diverse pathologic settings. Adv. Drug Deliv. Rev..

[20] Hori O., Brett J., Slattery T., Cao R., Zhang J., Chen J.X., Nagashima M., Lundh E.R., Vijay S., Nitecki D. (1995). The receptor for advanced glycation end products (RAGE) is a cellular binding site for amphoterin. Mediation of neurite outgrowth and co-expression of rage and amphoterin in the developing nervous system. J. Biol. Chem..

[21] Donato R. (2001). S100: A multigenic family of calcium-modulated proteins of the EF-hand type with intracellular and extracellular functional roles. Int. J. Biochem. Cell Biol..

[22] Schmidt A.M., Yan S.D., Yan S.F., Stern D.M. (2001). The multiligand receptor RAGE as a progression factor amplifying immune and inflammatory responses. J. Clin. Investig..

[23] Bierhaus A., Humpert P.M., Morcos M., Wendt T., Chavakis T., Arnold B., Stern D.M., Nawroth P.P. (2005). Understanding RAGE, the receptor for advanced glycation end products. J. Mol. Med..

[24] Donato R. (2007). RAGE: A single receptor for several ligands and different cellular responses: The case of certain S100 proteins. Curr. Mol. Med..

[25] Yan S.F., Ramasamy R., Schmidt A.M. (2010). The RAGE axis: A fundamental mechanism signaling danger to the vulnerable vasculature. Circ. Res..

[26] Sims G.P., Rowe D.C., Rietdijk S.T., Herbst R., Coyle A.J. (2010). HMGB1 and RAGE in inflammation and cancer. Annu. Rev. Immunol..

[27] D’Agati V., Schmidt A.M. (2010). RAGE and the pathogenesis of chronic kidney disease. Nat. Rev. Nephrol..

[28] Deane R., Du Yan S., Submamaryan R.K., LaRue B., Jovanovic S., Hogg E., Welch D., Manness L., Lin C., Yu J. (2003). RAGE mediates amyloid-β peptide transport across the blood-brain barrier and accumulation in brain. Nat. Med..

[29] Kamide T., Kitao Y., Takeichi T., Okada A., Mohri H., Schmidt A.M., Kawano T., Munesue S., Yamamoto Y., Yamamoto H. (2012). RAGE mediates vascular injury and inflammation after global cerebral ischemia. Neurochem. Int..

[30] Jin Q., Chen H., Luo A., Ding F., Liu Z. (2011). S100A14 stimulates cell proliferation and induces cell apoptosis at different concentrations via receptor for advanced glycation end products (RAGE). PLoS One.

[31] Tator C.H., Fehlings M.G. (1991). Review of the secondary injury theory of acute spinal cord trauma with emphasis on vascular mechanisms. J. Neurosurg..

[32] Dumont R.J., Verma S., Okonkwo D.O., Hurlbert R.J., Boulos P.T., Ellegala D.B., Dumont A.S. (2001). Acute spinal cord injury, part II: Contemporary pharmacotherapy. Clin. Neuropharmacol..

[33] Whalley K., O’Neill P., Ferretti P. (2006). Changes in response to spinal cord injury with development: Vascularization, hemorrhage and apoptosis. Neuroscience.

[34] McDonald J.W., Sadowsky C. (2002). Spinal-cord injury. Lancet.

[35] Rosenfeld J.V., Bandopadhayay P., Goldschlager T., Brown D.J. (2008). The ethics of the treatment of spinal cord injury: Stem cell transplants, motor neuroprosthetics, and social equity. Top. Spinal Cord Inj. Rehabil..

[36] Genovese T., Esposito E., Mazzon E., di Paola R., Caminiti R., Bramanti P., Cappelani A., Cuzzocrea S. (2009). Absence of endogenous interleukin-10 enhances secondary inflammatory process after spinal cord compression injury in mice. J. Neurochem..

[37] Shen L.F., Cheng H., Tsai M.C., Kuo H.S., Chak K.F. (2009). PAL31 may play an important role as inflammatory modulator in the repair process of the spinal cord injury rat. J. Neurochem..

[38] Pineau I., Lacroix S. (2007). Proinflammatory cytokine synthesis in the injured mouse spinal cord: Multiphasic expression pattern and identification of the cell types involved. J. Comp. Neurol..

[39] Yang L., Jones N.R., Blumbergs P.C., van den Heuvel C., Moore E.J., Manavis J., Sarvestani G.T., Ghabriel M.N. (2005). Severity-dependent expression of pro-inflammatory cytokines in traumatic spinal cord injury in the rat. J. Clin. Neurosci..

[40] Yang L., Blumbergs P.C., Jones N.R., Manavis J., Sarvestani G.T., Ghabriel M.N. (2004). Early expression and cellular localization of proinflammatory cytokines interleukin-1β, interleukin-6, and tumor necrosis factor-α in human traumatic spinal cord injury. Spine.

[41] Schnell L., Fearn S., Schwab M.E., Perry V.H., Anthony D.C. (1999). Cytokine-induced acute inflammation in the brain and spinal cord. J. Neuropathol. Exp. Neurol..

[42] Cai Z.H., Song L.S., Gao C.P., Wu L.T., Qiu L.H., Xiang J.H. (2003). Cloning and expression of tumor necrosis factor (TNFα) cDNA from red seabream pagrus major. ShengWu HuaXue Yu ShengWu WuLi XueBao.

[43] Hermann G.E., Rogers R.C., Bresnahan J.C., Beattie M.S. (2001). Tumor necrosis factor-α induces cFOS and strongly potentiates glutamate-mediated cell death in the rat spinal cord. Neurobiol. Dis..

[44] Lee Y.B., Yune T.Y., Baik S.Y., Shin Y.H., Du S., Rhim H., Lee E.B., Kim Y.C., Shin M.L., Markelonis G.J. (2000). Role of tumor necrosis factor-alpha in neuronal and glial apoptosis after spinal cord injury. Exp. Neurol..

[45] Lu K.T., Wang Y.W., Yang J.T., Yang Y.L., Chen H.I. (2005). Effect of interleukin-1 on traumatic brain injury-induced damage to hippocampal neurons. J. Neurotrauma.

[46] Shamash S., Reichert F., Rotshenker S. (2002). The cytokine network of Wallerian degeneration: Tumor necrosis factor-α, interleukin-1α, and interleukin-1β. J. Neurosci..

[47] Streit W.J., Semple-Rowland S.L., Hurley S.D., Miller R.C., Popovich P.G., Stokes B.T. (1998). Cytokine mRNA profiles in contused spinal cord and axotomized facial nucleus suggest a beneficial role for inflammation and gliosis. Exp. Neurol..

[48] Dusart I., Schwab M.E. (1994). Secondary cell death and the inflammatory reaction after dorsal hemisection of the rat spinal cord. Eur. J. Neurosci..

[49] Fujiki M., Zhang Z., Guth L., Steward O. (1996). Genetic influences on cellular reactions to spinal cord injury: Activation of macrophages/microglia and astrocytes is delayed in mice carrying a mutation (WldS) that causes delayed Wallerian degeneration. J. Comp. Neurol..

[50] Silver J., Miller J.H. (2004). Regeneration beyond the glial scar. Nat. Rev. Neurosci..

[51] Schwab M.E. (2002). Repairing the injured spinal cord. Science.

[52] Yuan Y.M., He C. (2013). The glial scar in spinal cord injury and repair. Neurosci. Bull..

[53] Gensel J.C., Nakamura S., Guan Z., van Rooijen N., Ankeny D.P., Popovich P.G. (2009). Macrophages promote axon regeneration with concurrent neurotoxicity. J. Neurosci..

[54] Anthes D.L., Theriault E., Tator C.H. (1995). Characterization of axonal ultrastructural pathology following experimental spinal cord compression injury. Brain Res..

[55] Muradov J.M., Hagg T. (2013). Intravenous infusion of magnesium chloride improves epicenter blood flow during the acute stage of contusive spinal cord injury in rats. J. Neurotrauma.

[56] Tator C.H., Koyanagi I. (1997). Vascular mechanisms in the pathophysiology of human spinal cord injury. J. Neurosurg..

[57] Schmidt A.M., Stern D.M. (2000). RAGE: A new target for the prevention and treatment of the vascular and inflammatory complications of diabetes. Trends Endocrinol. Metab..

[58] Freigang J., Proba K., Leder L., Diederichs K., Sonderegger P., Welte W. (2000). The crystal structure of the ligand binding module of axonin-1/TAG-1 suggests a zipper mechanism for neural cell adhesion. Cell.

[59] Soroka V., Kolkova K., Kastrup J.S., Diederichs K., Breed J., Kiselyov V.V., Poulsen F.M., Larsen I.K., Welte W., Berezin V. (2003). Structure and interactions of NCAM Ig1-2-3 suggest a novel zipper mechanism for homophilic adhesion. Structure.

[60] Ramasamy R., Vannucci S.J., Yan S.S., Herold K., Yan S.F., Schmidt A.M. (2005). Advanced glycation end products and RAGE: A common thread in aging, diabetes, neurodegeneration, and inflammation. Glycobiology.

[61] Neeper M., Schmidt A.M., Brett J., Yan S.D., Wang F., Pan Y.C., Elliston K., Stern D., Shaw A. (1992). Cloning and expression of a cell surface receptor for advanced glycosylation end products of proteins. J. Biol. Chem..

[62] Yamamoto Y., Kato I., Doi T., Yonekura H., Ohashi S., Takeuchi M., Watanabe T., Yamagishi S., Sakurai S., Takasawa S. (2001). Development and prevention of advanced diabetic nephropathy in RAGE-overexpressing mice. J. Clin. Investig..

[63] Penfold S.A., Coughlan M.T., Patel S.K., Srivastava P.M., Sourris K.C., Steer D., Webster D.E., Thomas M.C., MacIsaac R.J., Jerums G. (2010). Circulating high-molecular-weight RAGE ligands activate pathways implicated in the development of diabetic nephropathy. Kidney Int..

[64] Gebhardt C., Riehl A., Durchdewald M., Nemeth J., Furstenberger G., Muller-Decker K., Enk A., Arnold B., Bierhaus A., Nawroth P.P. (2008). RAGE signaling sustains inflammation and promotes tumor development. J. Exp. Med..

[65] Riehl A., Nemeth J., Angel P., Hess J. (2009). The receptor RAGE: Bridging inflammation and cancer. Cell Commun. Signal..

[66] Huttunen H.J., Fages C., Rauvala H. (1999). Receptor for advanced glycation end products (RAGE)-mediated neurite outgrowth and activation of NF-κB require the cytoplasmic domain of the receptor but different downstream signaling pathways. J. Biol. Chem..

[67] Taguchi A., Blood D.C., del Toro G., Canet A., Lee D.C., Qu W., Tanji N., Lu Y., Lalla E., Fu C. (2000). Blockade of RAGE-amphoterin signalling suppresses tumour growth and metastases. Nature.

[68] Fages C., Nolo R., Huttunen H.J., Eskelinen E., Rauvala H. (2000). Regulation of cell migration by amphoterin. J. Cell Sci..

[69] Van Beijnum J.R., Buurman W.A., Griffioen A.W. (2008). Convergence and amplification of toll-like receptor (TLR) and receptor for advanced glycation end products (RAGE) signaling pathways via high mobility group B1 (HMGB1). Angiogenesis.

[70] Hsieh H.L., Schafer B.W., Sasaki N., Heizmann C.W. (2003). Expression analysis of S100 proteins and RAGE in human tumors using tissue microarrays. Biochem. Biophys. Res. Commun..

[71] Yan S.D., Chen X., Fu J., Chen M., Zhu H., Roher A., Slattery T., Zhao L., Nagashima M., Morser J. (1996). RAGE and amyloid-beta peptide neurotoxicity in Alzheimer’s disease. Nature.

[72] Zlokovic B.V. (2008). New therapeutic targets in the neurovascular pathway in Alzheimer’s disease. Neurotherapeutics.

[73] Yan S.F., Ramasamy R., Schmidt A.M. (2010). Soluble RAGE: Therapy and biomarker in unraveling the RAGE axis in chronic disease and aging. Biochem. Pharmacol..

[74] Candela P., Gosselet F., Saint-Pol J., Sevin E., Boucau M.C., Boulanger E., Cecchelli R., Fenart L. (2010). Apical-to-basolateral transport of amyloid-β peptides through blood-brain barrier cells is mediated by the receptor for advanced glycation end-products and is restricted by P-glycoprotein. J. Alzheimer’s Dis..

[75] Hofmann M.A., Drury S., Fu C., Qu W., Taguchi A., Lu Y., Avila C., Kambham N., Bierhaus A., Nawroth P. (1999). RAGE mediates a novel proinflammatory axis: A central cell surface receptor for S100/calgranulin polypeptides. Cell.

[76] Leclerc E., Fritz G., Vetter S.W., Heizmann C.W. (2009). Binding of S100 proteins to RAGE: An update. Biochim. Biophys. Acta.

[77] Yan S.S., Wu Z.Y., Zhang H.P., Furtado G., Chen X., Yan S.F., Schmidt A.M., Brown C., Stern A., LaFaille J. (2003). Suppression of experimental autoimmune encephalomyelitis by selective blockade of encephalitogenic T-cell infiltration of the central nervous system. Nat. Med..

[78] Matsumoto S., Yoshida T., Murata H., Harada S., Fujita N., Nakamura S., Yamamoto Y., Watanabe T., Yonekura H., Yamamoto H. (2008). Solution structure of the variable-type domain of the receptor for advanced glycation end products: New insight into AGE-RAGE interaction. Biochemistry.

[79] Koch M., Chitayat S., Dattilo B.M., Schiefner A., Diez J., Chazin W.J., Fritz G. (2010). Structural basis for ligand recognition and activation of RAGE. Structure.

[80] Bianchi M.E., Manfredi A.A. (2007). High-mobility group box 1 (HMGB1) protein at the crossroads between innate and adaptive immunity. Immunol. Rev..

[81] Rauvala H., Rouhiainen A. (2007). RAGE as a receptor of HMGB1 (Amphoterin): Roles in health and disease. Curr. Mol. Med..

[82] Lander H.M., Tauras J.M., Ogiste J.S., Hori O., Moss R.A., Schmidt A.M. (1997). Activation of the receptor for advanced glycation end products triggers a p21(ras)-dependent mitogen-activated protein kinase pathway regulated by oxidant stress. J. Biol. Chem..

[83] Sakaguchi T., Yan S.F., Yan S.D., Belov D., Rong L.L., Sousa M., Andrassy M., Marso S.P., Duda S., Arnold B. (2003). Central role of RAGE-dependent neointimal expansion in arterial restenosis. J. Clin. Investig..

[84] Han S.H., Kim Y.H., Mook-Jung I. (2011). RAGE: The beneficial and deleterious effects by diverse mechanisms of actions. Mol. Cells.

[85] Hudson B.I., Kalea A.Z., del Mar Arriero M., Harja E., Boulanger E., D’Agati V., Schmidt A.M. (2008). Interaction of the RAGE cytoplasmic domain with diaphanous-1 is required for ligand-stimulated cellular migration through activation of Rac1 and Cdc42. J. Biol. Chem..

[86] Ramasamy R., Yan S.F., Schmidt A.M. (2009). RAGE: Therapeutic target and biomarker of the inflammatory response—The evidence mounts. J. Leukoc. Biol..

[87] Xu Y., Toure F., Qu W., Lin L., Song F., Shen X., Rosario R., Garcia J., Schmidt A.M., Yan S.F. (2010). Advanced glycation end product (AGE)-receptor for AGE (RAGE) signaling and up-regulation of Egr-1 in hypoxic macrophages. J. Biol. Chem..

[88] Bierhaus A., Nawroth P.P. (2009). Multiple levels of regulation determine the role of the receptor for AGE (RAGE) as common soil in inflammation, immune responses and diabetes mellitus and its complications. Diabetologia.

[89] Bierhaus A., Schiekofer S., Schwaninger M., Andrassy M., Humpert P.M., Chen J., Hong M., Luther T., Henle T., Kloting I. (2001). Diabetes-associated sustained activation of the transcription factor nuclear factor-κB. Diabetes.

[90] Bierhaus A., Stern D.M., Nawroth P.P. (2006). RAGE in inflammation: A new therapeutic target?. Curr. Opin. Investig. Drugs.

[91] Chen K.B., Uchida K., Nakajima H., Yayama T., Hirai T., Rodriguez Guerrero A., Kobayashi S., Ma W.Y., Liu S.Y., Zhu P. (2011). High-mobility group box-1 and its receptors contribute to proinflammatory response in the acute phase of spinal cord injury in rats. Spine.

[92] Kawabata H., Setoguchi T., Yone K., Souda M., Yoshida H., Kawahara K., Maruyama I., Komiya S. (2010). High mobility group box 1 is upregulated after spinal cord injury and is associated with neuronal cell apoptosis. Spine.

[93] Guo J.D., Li L., Shi Y.M., Wang H.D., Yuan Y.L., Shi X.X., Hou S.X. (2014). Genetic ablation of receptor for advanced glycation end products promotes functional recovery in mouse model of spinal cord injury. Mol. Cell. Biochem..

[94] Yonekura H., Yamamoto Y., Sakurai S., Petrova R.G., Abedin M.J., Li H., Yasui K., Takeuchi M., Makita Z., Takasawa S. (2003). Novel splice variants of the receptor for advanced glycation end-products expressed in human vascular endothelial cells and pericytes, and their putative roles in diabetes-induced vascular injury. Biochem. J..

[95] Xu D., Young J., Song D., Esko J.D. (2011). Heparan sulfate is essential for high mobility group protein 1 (HMGB1) signaling by the receptor for advanced glycation end products (RAGE). J. Biol. Chem..

[96] Kislinger T., Fu C., Huber B., Qu W., Taguchi A., Du Yan S., Hofmann M., Yan S.F., Pischetsrieder M., Stern D. (1999). *N*(epsilon)-(carboxymethyl)lysine adducts of proteins are ligands for receptor for advanced glycation end products that activate cell signaling pathways and modulate gene expression. J. Biol. Chem..

[97] Guazzi S., Strangio A., Franzi A.T., Bianchi M.E. (2003). HMGB1, an architectural chromatin protein and extracellular signalling factor, has a spatially and temporally restricted expression pattern in mouse brain. Gene Expr. Patterns.

[98] Enokido Y., Yoshitake A., Ito H., Okazawa H. (2008). Age-dependent change of HMGB1 and DNA double-strand break accumulation in mouse brain. Biochem. Biophys. Res. Commun..

[99] Daston M.M., Ratner N. (1994). Amphoterin (P30, HMG-1) and RIP are early markers of oligodendrocytes in the developing rat spinal cord. J. Neurocytol..

[100] Tenenbaum T., Essmann F., Adam R., Seibt A., Janicke R.U., Novotny G.E., Galla H.J., Schroten H. (2006). Cell death, caspase activation, and HMGB1 release of porcine choroid plexus epithelial cells during *Streptococcus suis* infection *in vitro*. Brain Res..

[101] Gao H.M., Zhou H., Zhang F., Wilson B.C., Kam W., Hong J.S. (2011). HMGB1 acts on microglia Mac1 to mediate chronic neuroinflammation that drives progressive neurodegeneration. J. Neurosci..

[102] Daston M.M., Ratner N. (1991). Expression of P30, a protein with adhesive properties, in Schwann cells and neurons of the developing and regenerating peripheral nerve. J. Cell Biol..

[103] Adami C., Sorci G., Blasi E., Agneletti A.L., Bistoni F., Donato R. (2001). S100B expression in and effects on microglia. Glia.

[104] Huttunen H.J., Kuja-Panula J., Sorci G., Agneletti A.L., Donato R., Rauvala H. (2000). Coregulation of neurite outgrowth and cell survival by amphoterin and S100 proteins through receptor for advanced glycation end products (RAGE) activation. J. Biol. Chem..

[105] Yan S.F., Ramasamy R., Schmidt A.M. (2008). Mechanisms of disease: Advanced glycation end-products and their receptor in inflammation and diabetes complications. Nat. Clin. Pract. Endocrinol. Metab..

[106] Meneghini V., Francese M.T., Carraro L., Grilli M. (2010). A novel role for the Receptor for Advanced Glycation End-products in neural progenitor cells derived from adult SubVentricular Zone. Mol. Cell. Neurosci..

[107] Liu X.Z., Xu X.M., Hu R., Du C., Zhang S.X., McDonald J.W., Dong H.X., Wu Y.J., Fan G.S., Jacquin M.F. (1997). Neuronal and glial apoptosis after traumatic spinal cord injury. J. Neurosci..

[108] Kwon B.K., Oxland T.R., Tetzlaff W. (2002). Animal models used in spinal cord regeneration research. Spine.

[109] Kwon B.K., Tetzlaff W., Grauer J.N., Beiner J., Vaccaro A.R. (2004). Pathophysiology and pharmacologic treatment of acute spinal cord injury. Spine J..

[110] Norenberg M.D., Smith J., Marcillo A. (2004). The pathology of human spinal cord injury: Defining the problems. J. Neurotrauma.

[111] Foote A.K., Blakemore W.F. (2005). Inflammation stimulates remyelination in areas of chronic demyelination. Brain.

[112] Ludwin S.K. (1980). Chronic demyelination inhibits remyelination in the central nervous system. An analysis of contributing factors. Lab. Investig..

[113] Miron V.E., Boyd A., Zhao J.W., Yuen T.J., Ruckh J.M., Shadrach J.L., van Wijngaarden P., Wagers A.J., Williams A., Franklin R.J. (2013). M2 microglia and macrophages drive oligodendrocyte differentiation during CNS remyelination. Nat. Neurosci..

[114] Morell P., Barrett C.V., Mason J.L., Toews A.D., Hostettler J.D., Knapp G.W., Matsushima G.K. (1998). Gene expression in brain during cuprizone-induced demyelination and remyelination. Mol. Cell. Neurosci..

[115] Katoh S., Ikata T., Tsubo M., Hamada Y., el Masry W.S. (1997). Possible implication of leukocytes in secondary pathological changes after spinal cord injury. Injury.

[116] Li Z., Hogan E.L., Banik N.L. (1996). Role of calpain in spinal cord injury: Increased calpain immunoreactivity in rat spinal cord after impact trauma. Neurochem. Res..

[117] Lu J., Ashwell K.W., Waite P. (2000). Advances in secondary spinal cord injury: Role of apoptosis. Spine.

[118] Habgood M.D., Bye N., Dziegielewska K.M., Ek C.J., Lane M.A., Potter A., Morganti-Kossmann C., Saunders N.R. (2007). Changes in blood-brain barrier permeability to large and small molecules following traumatic brain injury in mice. Eur. J. Neurosci..

[119] Han X., Lu M., Wang S., Lv D., Liu H. (2012). Targeting IKK/NF-κB pathway reduces infiltration of inflammatory cells and apoptosis after spinal cord injury in rats. Neurosci. Lett..

[120] Brambilla R., Bracchi-Ricard V., Hu W.H., Frydel B., Bramwell A., Karmally S., Green E.J., Bethea J.R. (2005). Inhibition of astroglial nuclear factor kappaB reduces inflammation and improves functional recovery after spinal cord injury. J. Exp. Med..

[121] Brambilla R., Persaud T., Hu X., Karmally S., Shestopalov V.I., Dvoriantchikova G., Ivanov D., Nathanson L., Barnum S.R., Bethea J.R. (2009). Transgenic inhibition of astroglial NF-κB improves functional outcome in experimental autoimmune encephalomyelitis by suppressing chronic central nervous system inflammation. J. Immunol..

[122] Yaser A.M., Huang Y., Zhou R.R., Hu G.S., Xiao M.F., Huang Z.B., Duan C.J., Tian W., Tang D.L., Fan X.G. (2012). The role of receptor for advanced glycation end products (RAGE) in the proliferation of hepatocellular carcinoma. Int. J. Mol. Sci..

[123] Huang Y., Xie K., Li J., Xu N., Gong G., Wang G., Yu Y., Dong H., Xiong L. (2011). Beneficial effects of hydrogen gas against spinal cord ischemia-reperfusion injury in rabbits. Brain Res..

[124] Wang Q., Ding Q., Zhou Y., Gou X., Hou L., Chen S., Zhu Z., Xiong L. (2009). Ethyl pyruvate attenuates spinal cord ischemic injury with a wide therapeutic window through inhibiting high-mobility group box 1 release in rabbits. Anesthesiology.

[125] Esposito E., Genovese T., Caminiti R., Bramanti P., Meli R., Cuzzocrea S. (2009). Melatonin reduces stress-activated/mitogen-activated protein kinases in spinal cord injury. J. Pineal Res..

[126] Arancio O., Zhang H.P., Chen X., Lin C., Trinchese F., Puzzo D., Liu S., Hegde A., Yan S.F., Stern A. (2004). RAGE potentiates Aβ-induced perturbation of neuronal function in transgenic mice. EMBO J..

[127] Brett J., Schmidt A.M., Yan S.D., Zou Y.S., Weidman E., Pinsky D., Nowygrod R., Neeper M., Przysiecki C., Shaw A. (1993). Survey of the distribution of a newly characterized receptor for advanced glycation end products in tissues. Am. J. Pathol..

[128] Vincent A.M., Perrone L., Sullivan K.A., Backus C., Sastry A.M., Lastoskie C., Feldman E.L. (2007). Receptor for advanced glycation end products activation injures primary sensory neurons via oxidative stress. Endocrinology.

[129] Bonaldi T., Talamo F., Scaffidi P., Ferrera D., Porto A., Bachi A., Rubartelli A., Agresti A., Bianchi M.E. (2003). Monocytic cells hyperacetylate chromatin protein HMGB1 to redirect it towards secretion. EMBO J..

[130] Scaffidi P., Misteli T., Bianchi M.E. (2002). Release of chromatin protein HMGB1 by necrotic cells triggers inflammation. Nature.

[131] Pisetsky D.S., Erlandsson-Harris H., Andersson U. (2008). High-mobility group box protein 1 (HMGB1): An alarmin mediating the pathogenesis of rheumatic disease. Arthritis Res. Ther..

[132] Dumitriu I.E., Baruah P., Valentinis B., Voll R.E., Herrmann M., Nawroth P.P., Arnold B., Bianchi M.E., Manfredi A.A., Rovere-Querini P. (2005). Release of high mobility group box 1 by dendritic cells controls T cell activation via the receptor for advanced glycation end products. J. Immunol..

[133] Kokkola R., Andersson A., Mullins G., Ostberg T., Treutiger C.J., Arnold B., Nawroth P., Andersson U., Harris R.A., Harris H.E. (2005). RAGE is the major receptor for the proinflammatory activity of HMGB1 in rodent macrophages. Scand. J. Immunol..

[134] Andersson A., Covacu R., Sunnemark D., Danilov A.I., dal Bianco A., Khademi M., Wallstrom E., Lobell A., Brundin L., Lassmann H. (2008). Pivotal advance: HMGB1 expression in active lesions of human and experimental multiple sclerosis. J. Leukoc. Biol..

[135] Lin L. (2006). RAGE on the Toll Road?. Cell Mol. Immunol..

[136] Ruan B.H., Li X., Winkler A.R., Cunningham K.M., Kuai J., Greco R.M., Nocka K.H., Fitz L.J., Wright J.F., Pittman D.D. (2010). Complement C3a, CpG oligos, and DNA/C3a complex stimulate IFN-α production in a receptor for advanced glycation end product-dependent manner. J. Immunol..

[137] Yamamoto Y., Harashima A., Saito H., Tsuneyama K., Munesue S., Motoyoshi S., Han D., Watanabe T., Asano M., Takasawa S. (2011). Septic shock is associated with receptor for advanced glycation end products ligation of LPS. J. Immunol..

[138] Kawabata D., Venkatesh J., Ramanujam M., Davidson A., Grimaldi C.M., Diamond B. (2010). Enhanced selection of high affinity DNA-reactive B cells following cyclophosphamide treatment in mice. PLoS One.

[139] Chen G., Ward M.F., Sama A.E., Wang H. (2004). Extracellular HMGB1 as a proinflammatory cytokine. J. Interferon Cytokine Res..

[140] Yang H., Wang H., Tracey K.J. (2001). HMG-1 rediscovered as a cytokine. Shock.

[141] Wang H., Yang H., Czura C.J., Sama A.E., Tracey K.J. (2001). HMGB1 as a late mediator of lethal systemic inflammation. Am. J. Respir. Crit. Care Med..

[142] Yang H., Wang H., Czura C.J., Tracey K.J. (2002). HMGB1 as a cytokine and therapeutic target. J. Endotoxin Res..

[143] Dumitriu I.E., Bianchi M.E., Bacci M., Manfredi A.A., Rovere-Querini P. (2007). The secretion of HMGB1 is required for the migration of maturing dendritic cells. J. Leukoc. Biol..

[144] Yang D., Chen Q., Yang H., Tracey K.J., Bustin M., Oppenheim J.J. (2007). High mobility group box-1 protein induces the migration and activation of human dendritic cells and acts as an alarmin. J. Leukoc. Biol..

[145] Messmer D., Yang H., Telusma G., Knoll F., Li J., Messmer B., Tracey K.J., Chiorazzi N. (2004). High mobility group box protein 1: An endogenous signal for dendritic cell maturation and Th1 polarization. J. Immunol..

[146] Rovere-Querini P., Capobianco A., Scaffidi P., Valentinis B., Catalanotti F., Giazzon M., Dumitriu I.E., Muller S., Iannacone M., Traversari C. (2004). HMGB1 is an endogenous immune adjuvant released by necrotic cells. EMBO Rep..

[147] Andersson U., Wang H., Palmblad K., Aveberger A.C., Bloom O., Erlandsson-Harris H., Janson A., Kokkola R., Zhang M., Yang H. (2000). High mobility group 1 protein (HMG-1) stimulates proinflammatory cytokine synthesis in human monocytes. J. Exp. Med..

[148] Park J.S., Gamboni-Robertson F., He Q., Svetkauskaite D., Kim J.Y., Strassheim D., Sohn J.W., Yamada S., Maruyama I., Banerjee A. (2006). High mobility group box 1 protein interacts with multiple Toll-like receptors. Am. J. Physiol. Cell Physiol..

[149] Park J.S., Svetkauskaite D., He Q., Kim J.Y., Strassheim D., Ishizaka A., Abraham E. (2004). Involvement of toll-like receptors 2 and 4 in cellular activation by high mobility group box 1 protein. J. Biol. Chem..

[150] Park J.S., Arcaroli J., Yum H.K., Yang H., Wang H., Yang K.Y., Choe K.H., Strassheim D., Pitts T.M., Tracey K.J. (2003). Activation of gene expression in human neutrophils by high mobility group box 1 protein. Am. J. Physiol. Cell Physiol..

[151] Vajn K., Plunkett J.A., Tapanes-Castillo A., Oudega M. (2013). Axonal regeneration after spinal cord injury in zebrafish and mammals: Differences, similarities, translation. Neurosci. Bull..

[152] Cao Q., Xu X.M., Devries W.H., Enzmann G.U., Ping P., Tsoulfas P., Wood P.M., Bunge M.B., Whittemore S.R. (2005). Functional recovery in traumatic spinal cord injury after transplantation of multineurotrophin-expressing glial-restricted precursor cells. J. Neurosci..

[153] Cummings B.J., Uchida N., Tamaki S.J., Salazar D.L., Hooshmand M., Summers R., Gage F.H., Anderson A.J. (2005). Human neural stem cells differentiate and promote locomotor recovery in spinal cord-injured mice. Proc. Natl. Acad. Sci. USA.

[154] Hofstetter C.P., Holmstrom N.A., Lilja J.A., Schweinhardt P., Hao J., Spenger C., Wiesenfeld-Hallin Z., Kurpad S.N., Frisen J., Olson L. (2005). Allodynia limits the usefulness of intraspinal neural stem cell grafts; directed differentiation improves outcome. Nature Neurosci..

[155] Karimi-Abdolrezaee S., Eftekharpour E., Wang J., Morshead C.M., Fehlings M.G. (2006). Delayed transplantation of adult neural precursor cells promotes remyelination and functional neurological recovery after spinal cord injury. J. Neurosci..

[156] Lee K.H., Yoon D.H., Park Y.G., Lee B.H. (2005). Effects of glial transplantation on functional recovery following acute spinal cord injury. J. Neurotrauma.

[157] Mitsui T., Shumsky J.S., Lepore A.C., Murray M., Fischer I. (2005). Transplantation of neuronal and glial restricted precursors into contused spinal cord improves bladder and motor functions, decreases thermal hypersensitivity, and modifies intraspinal circuitry. J. Neurosci..

[158] Rauvala H., Pihlaskari R. (1987). Isolation and some characteristics of an adhesive factor of brain that enhances neurite outgrowth in central neurons. J. Biol. Chem..

[159] Zhao Z., Nair S.M., Chou D.K., Tobet S.A., Jungalwala F.B. (2000). Expression and role of sulfoglucuronyl (HNK-1) carbohydrate and its binding protein SBP-1 in developing rat cerebral cortex. J. Neurosci. Res..

[160] Huttunen H.J., Rauvala H. (2004). Amphoterin as an extracellular regulator of cell motility: From discovery to disease. J. Intern. Med..

[161] Huttunen H.J., Kuja-Panula J., Rauvala H. (2002). Receptor for advanced glycation end products (RAGE) signaling induces CREB-dependent chromogranin expression during neuronal differentiation. J. Biol. Chem..

[162] Srikrishna G., Huttunen H.J., Johansson L., Weigle B., Yamaguchi Y., Rauvala H., Freeze H.H. (2002). *N*-Glycans on the receptor for advanced glycation end products influence amphoterin binding and neurite outgrowth. J. Neurochem..

[163] Rauvala H., Huttunen H.J., Fages C., Kaksonen M., Kinnunen T., Imai S., Raulo E., Kilpelainen I. (2000). Heparin-binding proteins HB-GAM (pleiotrophin) and amphoterin in the regulation of cell motility. Matrix Biol..

[164] Reddy M.A., Li S.L., Sahar S., Kim Y.S., Xu Z.G., Lanting L., Natarajan R. (2006). Key role of Src kinase in S100B-induced activation of the receptor for advanced glycation end products in vascular smooth muscle cells. J. Biol. Chem..

[165] Riuzzi F., Sorci G., Donato R. (2006). The amphoterin (HMGB1)/receptor for advanced glycation end products (RAGE) pair modulates myoblast proliferation, apoptosis, adhesiveness, migration, and invasiven. Functional inactivation of RAGE in L6 myoblasts results in tumor formation *in vivo*. J. Biol. Chem..

[166] Chavakis E., Hain A., Vinci M., Carmona G., Bianchi M.E., Vajkoczy P., Zeiher A.M., Chavakis T., Dimmeler S. (2007). High-mobility group box 1 activates integrin-dependent homing of endothelial progenitor cells. Circ. Res..

[167] Orlova V.V., Choi E.Y., Xie C., Chavakis E., Bierhaus A., Ihanus E., Ballantyne C.M., Gahmberg C.G., Bianchi M.E., Nawroth P.P. (2007). A novel pathway of HMGB1-mediated inflammatory cell recruitment that requires Mac-1-integrin. EMBO J..

[168] Saleh A., Smith D.R., Tessler L., Mateo A.R., Martens C., Schartner E., van der Ploeg R., Toth C., Zochodne D.W., Fernyhough P. (2013). Receptor for advanced glycation end-products (RAGE) activates divergent signaling pathways to augment neurite outgrowth of adult sensory neurons. Exp. Neurol..

[169] Bhattacharyya A., Oppenheim R.W., Prevette D., Moore B.W., Brackenbury R., Ratner N. (1992). S100 is present in developing chicken neurons and Schwann cells and promotes motor neuron survival *in vivo*. J. Neurobiol..

[170] Haglid K.G., Yang Q., Hamberger A., Bergman S., Widerberg A., Danielsen N. (1997). S-100β stimulates neurite outgrowth in the rat sciatic nerve grafted with acellular muscle transplants. Brain Res..

[171] Alexanian A.R., Bamburg J.R. (1999). Neuronal survival activity of s100betabeta is enhanced by calcineurin inhibitors and requires activation of NF-κB. FASEB J..

[172] Wu Y.Y., Bradshaw R.A. (1996). Induction of neurite outgrowth by interleukin-6 is accompanied by activation of Stat3 signaling pathway in a variant PC12 cell (E2) line. J. Biol. Chem..

[173] Wu C.L., Chou Y.H., Chang Y.J., Teng N.Y., Hsu H.L., Chen L. (2012). Interplay between cell migration and neurite outgrowth determines SH2B1β-enhanced neurite regeneration of differentiated PC12 cells. PLoS One.

[174] Woszczycka-Korczynska I., Olakowska E., Marcol W., Lewin-Kowalik J., Jedrzejowska-Szypulka H. (2013). Schwann cells in therapy of spinal cord injuries. Postepy Higieny i Medycyny Doswiadczalnej.

[175] Ozdemir M., Attar A., Kuzu I. (2012). Regenerative treatment in spinal cord injury. Curr. Stem Cell Res. Ther..

[176] Zawadzka M., Rivers L.E., Fancy S.P., Zhao C., Tripathi R., Jamen F., Young K., Goncharevich A., Pohl H., Rizzi M. (2010). CNS-resident glial progenitor/stem cells produce Schwann cells as well as oligodendrocytes during repair of CNS demyelination. Cell Stem Cell.

[177] Biernaskie J., Sparling J.S., Liu J., Shannon C.P., Plemel J.R., Xie Y., Miller F.D., Tetzlaff W. (2007). Skin-derived precursors generate myelinating Schwann cells that promote remyelination and functional recovery after contusion spinal cord injury. J. Neurosci..

[178] Bunge R.P., Puckett W.R., Becerra J.L., Marcillo A., Quencer R.M. (1993). Observations on the pathology of human spinal cord injury. A review and classification of 22 new cases with details from a case of chronic cord compression with extensive focal demyelination. Adv. Neurol..

[179] Plemel J.R., Duncan G., Chen K.W., Shannon C., Park S., Sparling J.S., Tetzlaff W. (2008). A graded forceps crush spinal cord injury model in mice. J. Neurotrauma.

[180] Wiliams R.R., Bunge M.B. (2012). Schwann cell transplantation: A repair strategy for spinal cord injury?. Prog. Brain Res..

[181] Akassoglou K., Yu W.M., Akpinar P., Strickland S. (2002). Fibrin inhibits peripheral nerve remyelination by regulating Schwann cell differentiation. Neuron.

[182] Rong L.L., Trojaborg W., Qu W., Kostov K., Yan S.D., Gooch C., Szabolcs M., Hays A.P., Schmidt A.M. (2004). Antagonism of RAGE suppresses peripheral nerve regeneration. FASEB J..

[183] Rong L.L., Yan S.F., Wendt T., Hans D., Pachydaki S., Bucciarelli L.G., Adebayo A., Qu W., Lu Y., Kostov K. (2004). RAGE modulates peripheral nerve regeneration via recruitment of both inflammatory and axonal outgrowth pathways. FASEB J..

[184] Dobrowsky R.T., Rouen S., Yu C. (2005). Altered neurotrophism in diabetic neuropathy: Spelunking the caves of peripheral nerve. J. Pharmacol. Exp. Ther..

[185] Perrone L., Peluso G., Melone M.A. (2008). RAGE recycles at the plasma membrane in S100B secretory vesicles and promotes Schwann cells morphological changes. J. Cell. Physiol..

[186] Sbai O., Devi T.S., Melone M.A., Feron F., Khrestchatisky M., Singh L.P., Perrone L. (2010). RAGE-TXNIP axis is required for S100B-promoted Schwann cell migration, fibronectin expression and cytokine secretion. J. Cell Sci..

